# Valorization of Silkworm Cocoons into Sustainable Nanomaterials: Applications and Challenges

**DOI:** 10.1021/acsbiomaterials.6c00221

**Published:** 2026-04-03

**Authors:** Caroliny M. Santos, Thiago F. Santos, Shuchi Tiwari, Arnóbio Antônio da Silva Júnior, J. H. O. Nascimento

**Affiliations:** † Postgraduate Program in Chemical Engineering, Federal University of Rio Grande do Norte, 59072-970 Natal, Rio Grande do Norte, Brazil; ‡ GPIMN—Micro and Nanotechnologies Innovation Research Group, Federal University of Rio Grande do Norte, 59072-970 Natal, Rio Grande do Norte, Brazil; § Department of Chemistry, 471717 VNR Vignana Jyothi College of Engineering and Technology, Hyderabad, Telangana 500090, India; ∥ Department of Pharmacy, Universidade Federal do Rio Grande do Norte (UFRN), 59012-570 Natal, Rio Grande do Norte, Brazil

**Keywords:** silk fibroin, nanoparticles, sustainability, biowaste, SDGs, green nanotechnology

## Abstract

Silk fibroin (SFb) derived from silkworm cocoons (*Bombyx mori*) is increasingly recognized as an innovative biodegradable protein for sustainable applications. We discuss its wide biomedical and industrial applications based on the review of 1791 articles from 2001 to 2025 (Scopus). There has been a large increase in research, with 66.44% of publications and 74.84% of citations during 2019–2025. SFb-based nanomaterials have excellent performance in tissue engineering (18.53%), drug delivery (60.59%), and in novel areas, including flexible electronics and sustainable construction, owing to their high strength, controllable structure, and optical transparency. Crucially, exploiting ∼50,000 tonnes of annual silk industry cocoon valorization for aerogels, hydrogels, and NPs promotes the circular economy and minimizes environmental impact using green technologies (lower kg CO_2_/kg fibroin). These developments make a direct, but by no means exclusive, contribution to the UN Sustainable Development Goals, in particular health (SDG 3), innovation (SDG 9), sustainable production (SDG 12), and climate action (SDG 13). Still, scaling, costs, and the long-term environmental consequences are hurdles. However, such concerns will need to be addressed through sustainable biomanufacturing and life-cycle assessments if SFb can be used to its full potential for a greener future.

## Graphical abstract

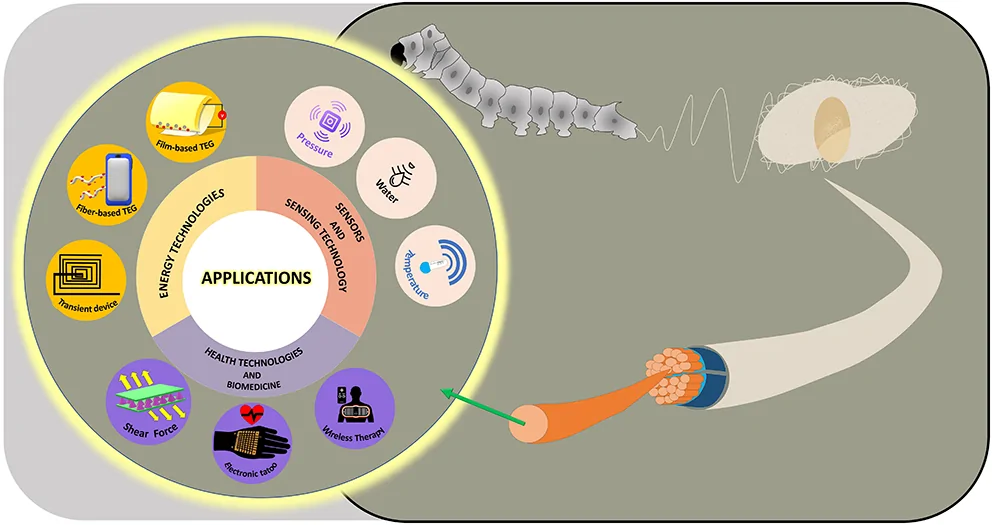

## 1 Introduction

The silk industry, one of the oldest economic activities of humanity, generates approximately 50,000 tons of waste annually (400,000 tons/year in dried cocoons), including sericin and discarded cocoons, which represent both an environmental challenge and an opportunity for sustainable valorization.^1,2^ Silkworm cocoon waste refers to discarded cocoons, defective fibers, and sericin-rich residues generated during silk processing. These byproducts represent an underutilized source of silk fibroin for sustainable nanomaterials. These residues, rich in silk fibroin, a biocompatible and biodegradable protein extracted from the cocoons of the silkworm (*Bombyx mori*), possess unique properties, including high mechanical strength, flexibility, and optical transparency, making them promising for the development of sustainable nanomaterials.^3,4^ The valorization of these byproducts aligns with the principles of the circular economy, reducing environmental impact and promoting sustainability, especially in the context of the UN Sustainable Development Goals (SDGs).^5–7^ The study of silk fibroin nanoparticles is justified by the need for alternatives to synthetic materials, often associated with high environmental impacts and dependence on nonrenewable resources.^8–10^ Due to its remarkable biocompatibility and biodegradability, fibroin has gained prominence in several cutting-edge applications, as shown in Figure 1. In the biomedical field, its main uses include drug delivery, which represents the largest application area (60.59%). This trend is driven by its ability to form stable nanoparticle systems for controlled release. And tissue engineering (scaffolds, 18.53%), where it serves as a matrix for cell regeneration. In addition, it is used in wound dressings (healing, 10.6%) and sensors (biodevices, 1.41%) thanks to its mechanical properties and ability to interact with biological tissues. In industrial contexts, fibroin has also been applied in storage (energy, 5.51%), building materials (2.35%), and electronics (1.45%), highlighting its versatility as a functional and sustainable material in emerging technologies.

**Figure 1:**
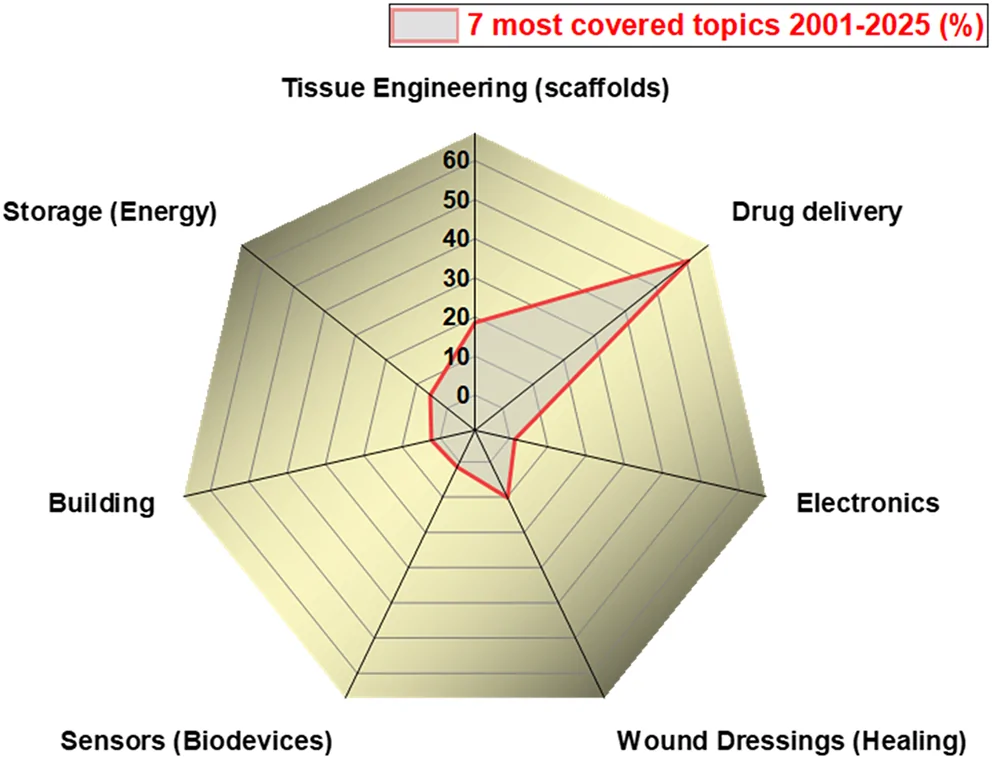
Distribution of major applications of silk fibroin-based nanomaterials (2001–2025).

The objective of this review is to critically evaluate the valorization of the silkworm cocoon as a sustainable source of silk fibroin for nanomaterial development, highlighting scientific advances, challenges, and future perspectives. The approach adopted is bibliometric-systematic, using the Scopus database to analyze 1791 articles published between 2001 and 2025, after refining criteria such as document type and language. This methodology allows identifying trends, gaps, and future perspectives in research, contributing to directing efforts in sustainable innovation and valorization for the silk industry.

## 2 Methodological Approach

The methodology used was bibliometric analysis, a widely accepted scientific method that applies mathematical and statistical techniques to evaluate scientific production.^11,12^ The study followed PRISMA guidelines, including identification, screening, eligibility, and inclusion steps, as shown in Figure 2. The research was developed based on Scopus, due to the greater variety of publications compared to the Web of Science.^13^ Data collection was carried out in March 2026, covering all scientific publications since the beginning of the historical period. The bibliometric methodology of this article follows a structured process for the retrieval and analysis of scientific data similar to methodologies in the current literature.^14–16^ It begins with the definition of the research framework and keywords, and strategy, performing a preliminary search in the Scopus database with the string (“silk fibroin OR fibroin OR silk”) AND (“cocoon waste OR silk waste OR discarded cocoon”) OR (“nanoparticle* OR nanofiber* OR hydrogel* OR drug delivery”) in the TITLE-ABS-KEY fields. After the initial search, the screening process was conducted in two stages: (i) title and abstract screening followed by (ii) full-text assessment. The inclusion criteria used were document type, language, and publication period (2001–2025). Performance Analysis evaluates aspects such as total publications, citations, countries, and keywords, while science mapping uses VOSviewer (version 1.6.19) with the following parameters: minimum occurrence of keywords = 20 (21 meet), normalization method = occurrences, clustering = 6 to identify thematic clusters.^17^ Content analysis examines the keywords and organizes the themes into clusters. Finally, the challenges and future perspectives stage suggests directions for future research.

**Figure 2:**
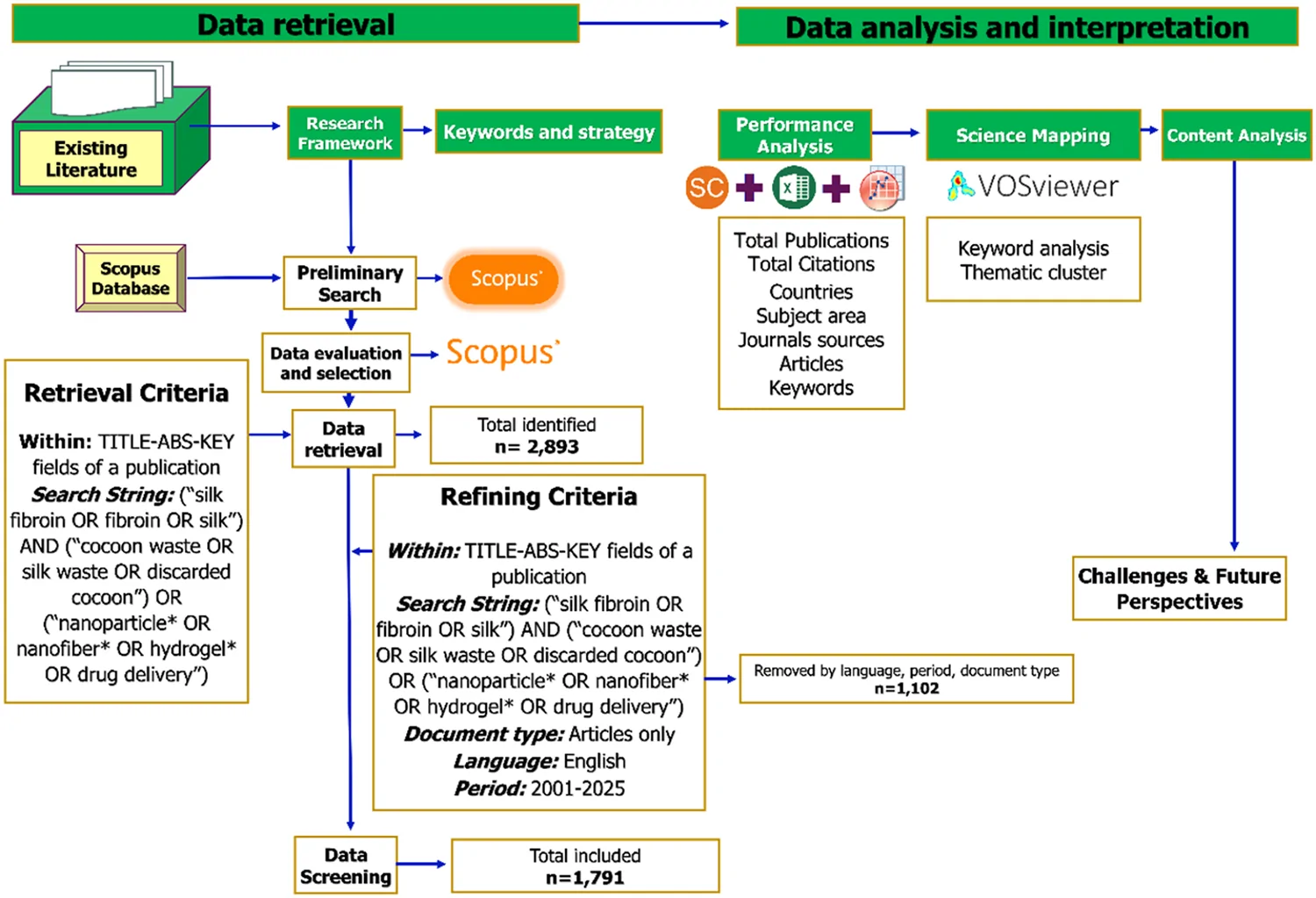
Bibliometric workflow for data retrieval and analysis. The process includes database search, screening, filtering, and data analysis steps following PRISMA guidelines to ensure reproducibility and transparency.

### 2.1 Inclusion/Exclusion Criteria

Articles were included based on relevance to silk fibroin nanomaterials document type (Articles), language (English), and publication period (2001–2025), resulting in a total of 1791 articles and exclusion criteria included conference papers, studies not directly related to fibroin nanomaterials, and articles lacking sufficient scientific detail (excluding 1102 articles).

### 2.2 Duplicate Removal

Duplicate records were identified and removed during the screening process.

## 3 Development and Scientific Overview of Fibroin Research

Analyzing the scientific production and citation data in Figure 3a, considering the time ranges (2001–2003, 2004–2006, 2007–2009, 2010–2012, 2013–2015, 2016–2018, 2019–2025), it was possible to observe the phased evolution in the development of fibroin research. In the first range, from 2001 to 2003, with only 0.95% published documents and 0.06% citations, the beginning of exploration in fibroin-based bionanomaterials was observed. Between 2004 and 2006, there have been 1.12% publications and 0.35% citations. The period from 2007 to 2009 marks the beginning of a consolidation phase, with 2.96% of documents and 1.45% of citations, indicating that some studies began to have greater visibility and influence on nanoparticles of fibroin from the cocoon of the silkworm caterpillar. In 2010–2012, with 5.58% of publications and 3.83% of citations, the volume and impact of research on silk protein materials increased. Between 2013 and 2015, there have been 9.55% of published documents and 7.58% of citations. The following period, 2016 to 2018, had 13.40% of publications and 11.88% of citations. From 2019 to 2025, there were 66.44% (1190) published documents and an impressive total of 74.84% (49,003) citations. This period reflects not only a massive increase in production but also a consolidated recognition of the importance and timeliness of fibroin-based nanomaterials, accounting for more than two-thirds of all accumulated production and impact in the analyzed period. Therefore, this division by bands highlights a clear trajectory of scientific maturation with three well-defined phases: an initial exploration phase (2001–2006), with more than 2% of publications; a consolidation phase (2007–2015), with volume and impact and academic recognition (2016–2025), responsible for more than 80% of publications and citations combined. Figure 3b complements this analysis by applying a second-degree polynomial model to fit the data and predict the future trend. The equation presented indicates an excellent fit of the model to the real data. Based on this projection, the number of publications could exceed 380 per year by 2032 if the current trend continues. Thus, this highlights not only consistent scientific production but also the consolidation and maturation of the emerging field of research, with expansion and increasing academic relevance.

**Figure 3:**
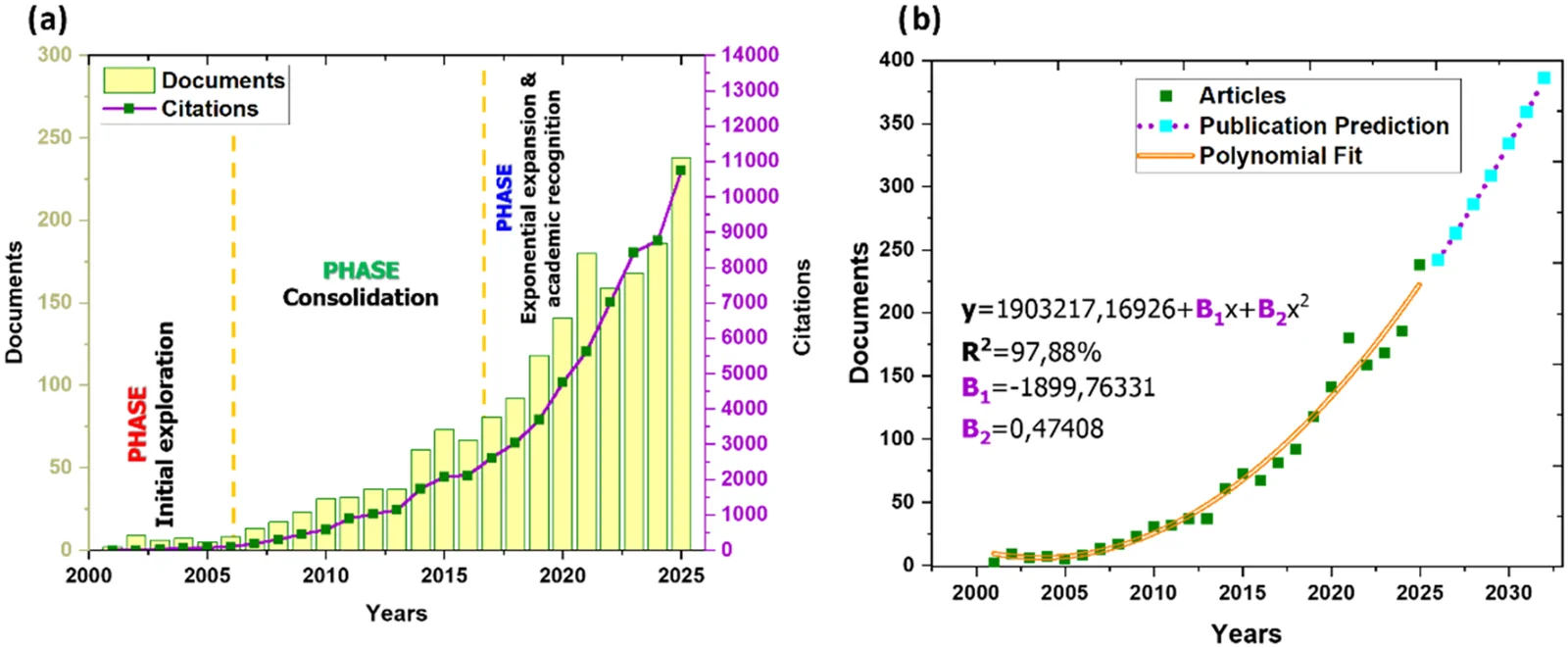
Trends in publications and citations of silk fibroin nanomaterials (2001–2025). (a) Annual number of documents and citations; (b) polynomial trend projection.

### 3.1 Key Countries

Figure 4 shows the evolution of scientific production by key areas of SFb-based nanomaterials between 2001 and 2025. Figure 4a shows the evolution of scientific production on SFb between 2001 and 2025, highlighting the countries that are most involved in research. China stands out significantly, especially from 2016–2018, with an exponential increase in the number of publications between 2019 and 2025, exceeding 560 articles. This reflects the country’s substantial investment in scientific research and technological innovation, particularly in biotechnology and sustainable materials. For the United States, too, there have been 169 publications in the latest period. Lower production countries, such as Iran and India, showed strong growth in scientific production after 2013. This increase is also reported by South Korea, the United Kingdom, and some European countries (Italy, Germany), although with more modest numbers. This reveals a significant global growth trend, especially in recent years, indicating an interest in sustainable nanomaterials and reflecting research priorities and strategic investments aimed at promoting solutions to environmental and health challenges. Figure 4b shows the % share of scientific production by country from 2016 to 2025 for SFb nanomaterials. China leads by a wide margin, accounting for 36.03% of publications, attributed to its strong silk industry, government investment in biomaterials, and established sericulture infrastructure.^18,19^ Followed by United States with 12.47%, India with 8.04%, and Iran with 5.76%. The United Kingdom (4.55%), South Korea (3.44%), and Thailand (3.1%) appear with moderate participation. Contributes 2.66% to Italy, 2.16% to Germany, and 2.00% to Japan. The dominance of China and the concentration of research in a few countries, despite a significant presence of other nations.

**Figure 4:**
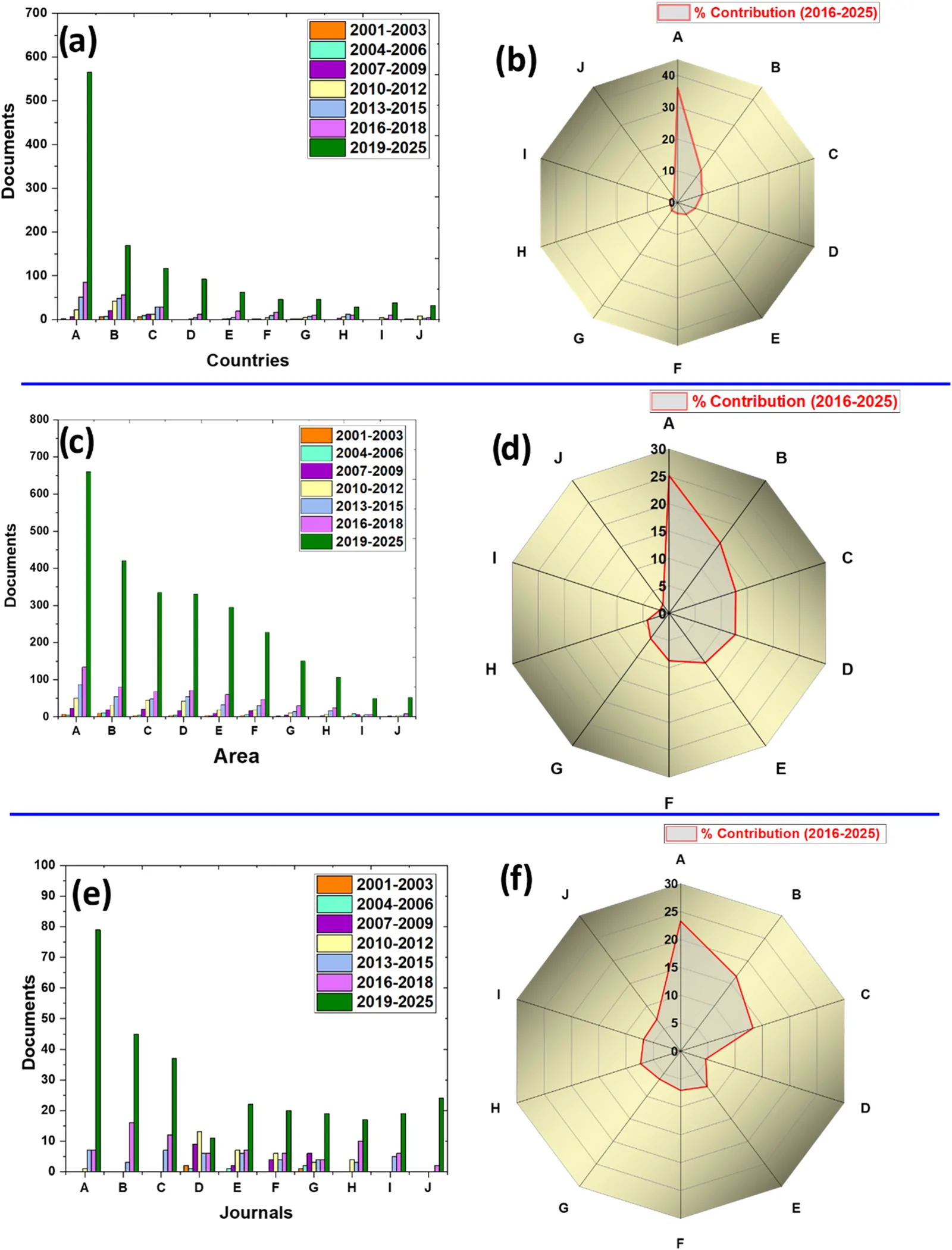
(a, b) Documents and % contribution by countries; (A) China; (B) United States; (C) India; (D) Iran; (E) United Kingdom; (F) South Korea; (G) Thailand; (H) Italy; (I) Germany; (J) Japan. (c, d) Documents and % contribution by areas; (A) Materials Science; (B) Engineering; (C) Chemical Engineering; (D) Biochemistry, Genetics and Molecular Biology; (E) Chemistry; (F) Pharmacology, Toxicology and Pharmaceutics; (G) Medicine; (H) Physics and Astronomy; (I) Agricultural and Biological Sciences; (J) Environmental Science; (e–f) Documents and % contribution by journals; (A) International Journal of Biological Macromolecules; (B) ACS Biomaterials Science and Engineering; (C) ACS Applied Materials and Interfaces; (D) Biomacromolecules; (E) Acta Biomaterialia; (F) International Journal of Pharmaceutics; (G) Journal of Drug Delivery Science and Technology; (H) Advanced Healthcare Materials; (I) Journal of Controlled Release; (J) Biomaterials.

### 3.2 Key Areas

Figure 4c shows the distribution of scientific publications from 2001 to 2025, highlighting the predominance of certain areas, especially Materials Science (A), which shows reaching 660 publications from 2019 to 2025. This reflects advances in research on sustainable materials, such as SFb nanomaterials, with applications in biotechnology and pharmacology. Areas such as Engineering (B) and Chemical Engineering (C) also show, with Engineering standing out in 2016–2018 with 80 publications, but without reaching the numbers of Materials Science. Areas such as Pharmacology, Toxicology, and Pharmaceutics (F), Medicine (G), and Physics & Astronomy (H) present more modest scientific production, with (F) reaching 228 publications from 2019 to 2025. Environmental Science (J) has a limited presence over time. Figure 4d shows the percentage distribution of publications on SFb nanomaterials. *Materials Science* leads with 25.15% of publications, followed by *Engineering* (15.84%), *Chemical Engineering* (12.77%), *Biochemistry*, *Genetics and Molecular Biology* (12.7%), and *Chemistry* (11.24%). *Pharmacology*, *Toxicology & Pharmaceutics* and *Medicine* demonstrate relevant involvement, with 8.71% and 5.73%, respectively, while *Physics & Astronomy* has a modest presence of 4.15%. The areas of *Environmental Science* (1.93%) and *Agricultural and Biological Sciences* (1.77%) have limited but growing contributions. This trend highlights the increasing multidisciplinarity of research focused on the development of sustainable materials and their applications in health and the environment.

### 3.3 Key Journals

Figure 4e shows the distribution of documents published in different journals between 2001 and 2025, highlighting the predominance of some journals. The *International Journal of Biological Macromolecules* (A) leads with 79 publications between 2019 and 2025, reflecting the growing focus on biocompatible materials such as SFb. The trend observed in other journals, such as that of *ACS Biomaterials Science and Engineering* (B), where 45 articles in the same period present relevant contributions, indicates the interest in sustainable biomaterials. *ACS Applied Materials and Interfaces* (C) also demonstrates an increase in research in biomedicine and materials engineering. Specific journals such as *Advanced Healthcare Materials* (H) and *Journal of Controlled Release* (I) have a smaller contribution, with a few publications. This shows that the potential of SFb is significant for the field, but to be in its early stages in some journals. There was a clear increase in publications from 2016 to 2018, reflecting the growing interest in sustainable materials. Figure 4f shows the percentage distribution of publications between 2016 and 2025. *International Journal of Biological Macromolecules (*A) leads with 23.31%, followed by *ACS Biomaterials Science* *& Engineering* (B) with 16.53% and *ACS Applied Materials* *& Interfaces* (C) with 13.28%. *Biomacromolecules* (D) and *Acta Biomaterialia* (E) complete the group with 7.86% and 7.32%, respectively. Other relevant journals include *Advanced Healthcare Materials* (H, 6.78%) and *Journal of Controlled Release* (I, 6.23%). The concentration of publications in high-impact journals reflects the scientific maturity and international relevance of SFb research.

### 3.4 Keyword Analysis

Figure 5 shows the distribution of publications on SFb and its derivatives between 2001 and 2025. The keyword ***“Silk Fibroin”*** (A) stands out, with going to 15.66% in 2019–2025, reflecting the increased interest in biotechnology and biomaterials. Publications on ***“Controlled Study”*** (B) also grew, reaching 10.18% in the last period, especially in medicine and pharmacology. While ***“Animals”*** (C) and ***“Nonhuman”*** (D) have 10.51% and 10.07%, reflecting the focus on safety and biocompatibility testing. This implies that the investigation of the biocompatibility of SFb-based materials and their potentiality in animal models is not only applicable to verify the safety and efficacy of these systems, but is also a prospective field of research because it is essential to demonstrate that these materials can be used safely in medical and biomedical applications. The keyword ***“Silk”*** (E) showed 8.38%. The keywords ***“Drug Delivery System”*** (F) and ***“Chemistry”*** (G) showed 8.92% and 9.05%, respectively. ***“Human”*** (H) followed ***“Controlled Drug Delivery”*** (I) and ***“Fibroins”*** (J), reflecting the increased interest in biotechnology and biomaterials, with 9.31%, 10.87%, and 8.06% of publications, respectively. The remarkable increase in publications reflects the growing consensus on the potential of SFb as a sustainable material for many applications in regenerative medicine, pharmacology, and nanotechnology. These percentages demonstrate SFb research over time and reveal an increased interest in fields related to its use in nanotechnology and biomedicine. The literature reveals a shift toward high-value biomedical applications, particularly in drug delivery and tissue engineering.^20–22^ Sustainable processing and the valorization of cocoon.^23–25^

**Figure 5:**
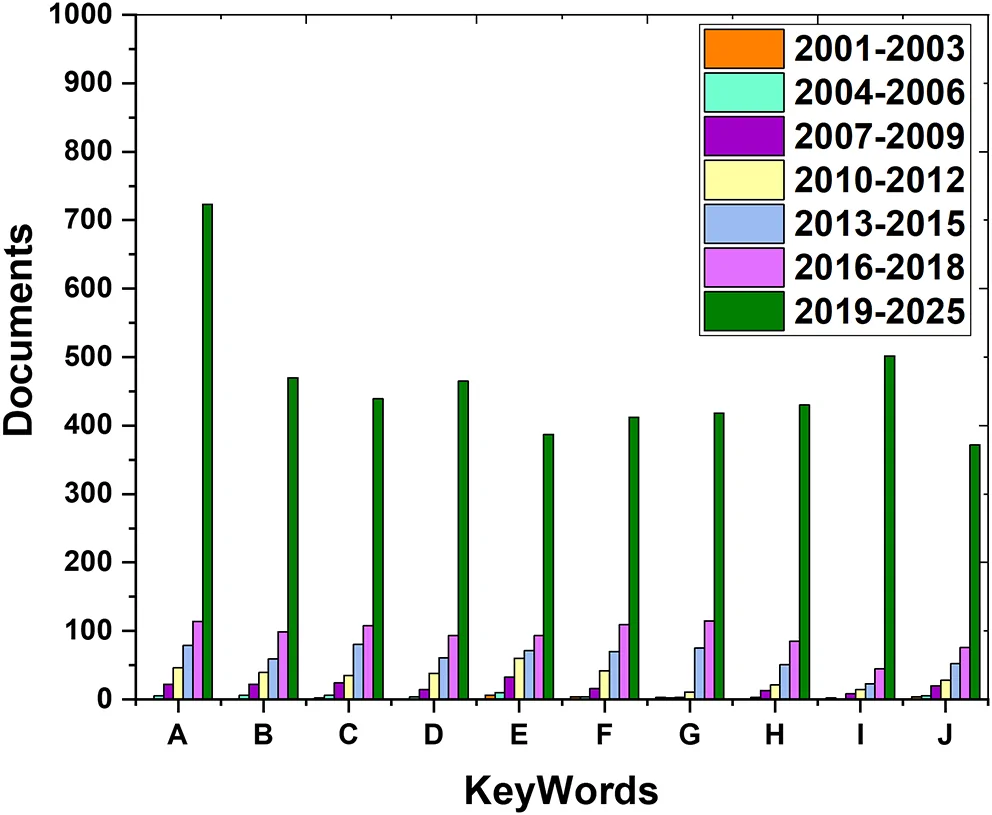
Documents published by keyword occurrences; (A) silk fibroin; (B) controlled study; (C) animals; (D) nonhuman; (E) silk; (F) drug delivery system; (G) chemistry; (H) human; (I) controlled drug delivery; (J) fibroins.

## 4 Composition and Structure

The silkworm, *Bombyx mori*, spins silk protein fibers naturally, and the fibroin fiber is produced via a secretory pathway, whereas sericin coats and encases the fiber. This protein is produced in the silkworms’ silk and makes up the silk fiber. It starts with the silkworm secreting the fibroin and sericin proteins through the spinning spindle from its salivary glands. When the fiber is extruded, fibroin microfibrils are assembled into an ordered material, providing the fiber with its tensile and other properties. In fact, this fiber at the molecular level, repeating units of glycine (Gly), sericin (Ser), and alanine (Ala), forms (Gly-Ala-Gly-Ala-Gly-Ser)*_n_*, as shown in Figure 6 for its chemical structure.

**Figure 6:**
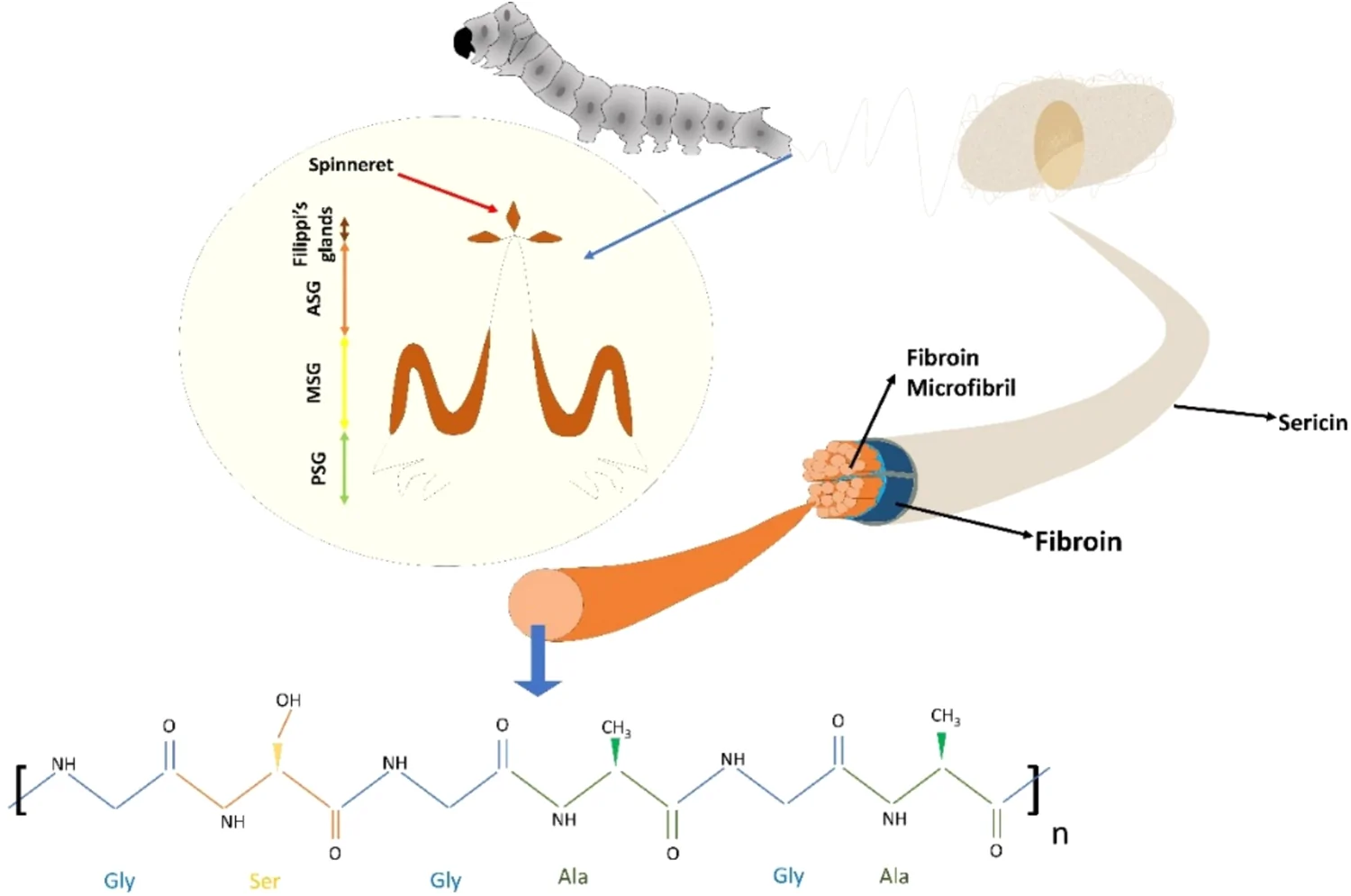
Silk fiber production process in *Bombyx mori*. The figure illustrates fibroin secretion and fiber formation, emphasizing the separation between fibroin and sericin, which is critical for cocoon valorization.

### 4.1 Chemical and Structural Properties of Silk Fibroin

Silk fibroin (SFb), a protein with repeating amino acid sequences (Gly, Ser, and Ala), possesses unique chemical and structural properties that support a variety of applications, as shown in Table 1. The primary structure of silk fibroin is mainly composed of repetitive sequences rich in glycine (Gly) and alanine (Ala), such as (Gly–Ala–Gly–Ala–Gly–Ser)*_n_*. Importantly, sericin is not part of the fibroin structure, but a distinct protein that surrounds fibroin fibers. Its biocompatibility allows integration with biological tissues and minimizes adverse reactions, making it suitable for tissue engineering and drug delivery.^26,27^ The biodegradability of SFb enables natural degradation with reduced environmental impact, ideal for dressings and biomedical devices.^28,29^ Its mechanical resistance, characterized by high strength and flexibility, supports its use in building materials and bone scaffolds.^30,31^ The tunability of its molecular structure facilitates customized modifications, enabling applications in flexible electronics,^32^ aerogel, and hydrogels.^33,34^ Additionally, its optical transparency is perfect for sensors and biodevices requiring photonic applications.^35,36^ These properties collectively highlight SFb’s versatility for technological and biomedical advancements.

**Table 1:** Chemical and Structural Properties of Silk Fibroin

properties	description	related applications	refs
Biocompatibility	Compatible with biological tissues, minimizes adverse reactions	Tissue engineering, drug delivery	^26^,^27^
Biodegradability	Degrades naturally, reduces environmental impact	Dressings, biomedical devices	^28^,^29^
Mechanical Resistance	High strength and flexibility	Building materials, bone scaffolds	^30^,^31^
Tunability	Molecular structure allows for customized modifications	Flexible electronics, Aerogels, and hydrogels	^33^,^34^,^37^
Optical	Optical transparency for photonic applications	Sensors, biodevices	^35^,^36^

## 5 Sustainability and Challenges

The growing demand for sustainable approaches in materials processing has intensified in response to the urgent need to preserve natural resources and mitigate the environmental impact of industrial production. In the field of biomaterials, this trend manifests itself in the effort to develop materials that combine biological performance with ecological responsibility, eliminating toxic substances, reducing energy consumption, and promoting more sustainable life-cycle processes. However, the relationship between “biomaterial” and “sustainability” is not always straightforward or guaranteed. Although biomaterials are defined by their biological applications, it is a misconception to assume that they are inherently environmentally friendly. Silk, for instance, is well-known for its fibroin, a biocompatible, biodegradable, and highly resistant protein, which illustrates this contradiction. While fibroin is highly valued for regenerative and medical applications, its extraction and transformation often involve environmentally harmful processes, such as the use of harsh solvents and significant energy consumption. Figure 7, which depicts the processing cycle of silk fibroin, highlights stages, such as synthesis and dissolution, during which the natural raw material is converted into moldable forms for various purposes. This cycle, however, also exposes a critical issue: the gap between the material’s “green” potential and the industrial practices required to realize it. Dissolved silk can be used in promising fields such as drug delivery, cosmetics, aerogels, hydrogels, and tissue engineering, but the true sustainability of these applications depends on re-evaluating the extraction, purification, and processing methods involved.^38–40^ While silk cocoons are the primary source, they can also be molded for a variety of purposes. The optimized silk processing cycle can reduce environmental impact while aligning with sustainability principles.

**Figure 7:**
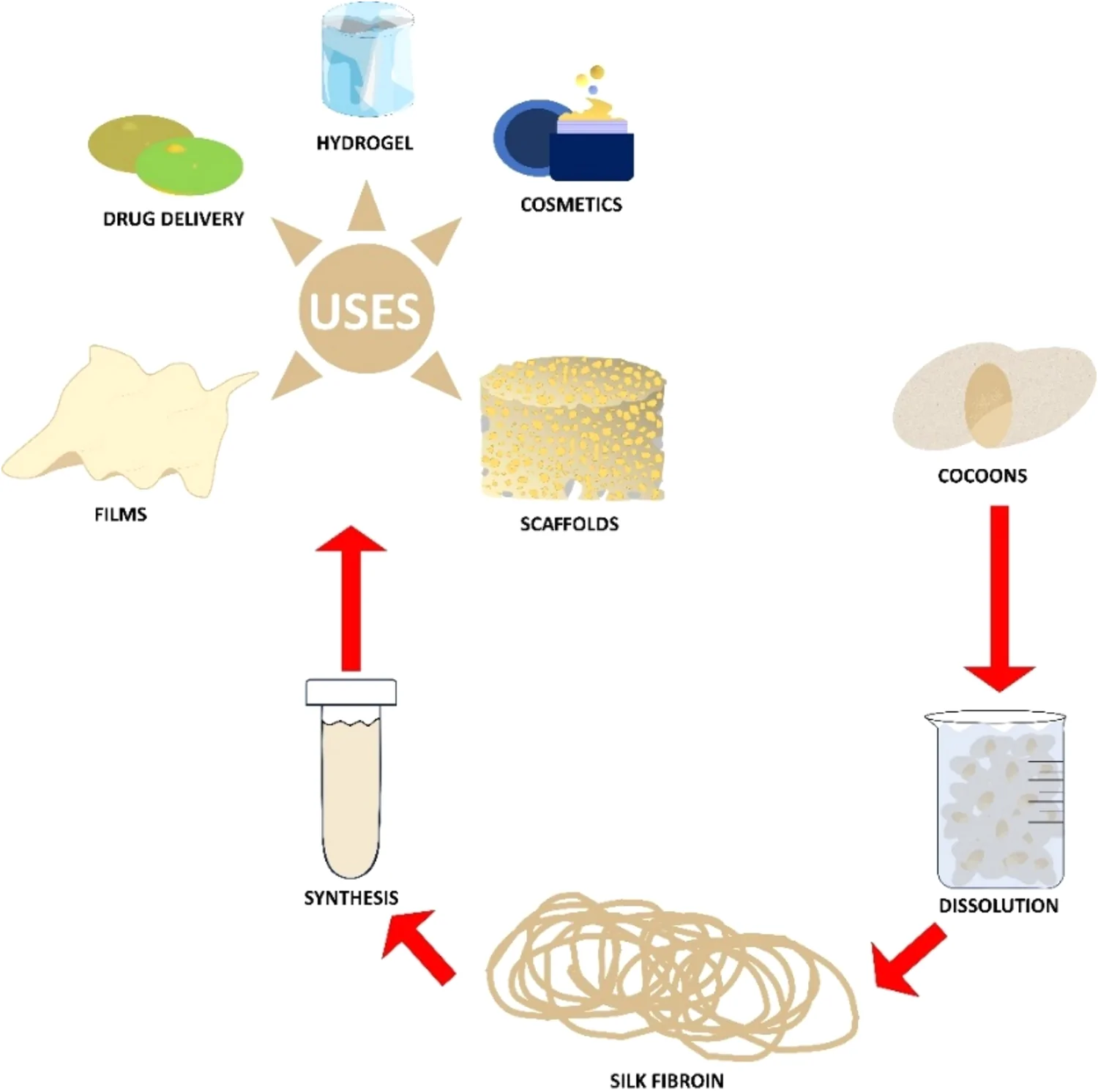
Overview of the silkworm life cycle and its relevance to fibroin extraction and biomedical applications.

The carbon footprint of silk processing varies considerably depending on energy demand, the type of chemical reagents used, and the overall resource intensity.^41^ The values reported in the literature vary widely. The carbon footprint of raw silk ranged from 2.41 to 28.56 kg of CO_2_e/kg (11.8×), with 89.1 to 96.5% of emissions linked to energy; allocation reduced the carbon footprint by 9.66–12.45%. Hotspots were drying (48.1%; 49.3%) and spinning (up to 69%). Emissions were dominated by Scope 2 (A) or Scope 1 (B–E), with CH_4_ and N_2_O being relevant. Electric heating is advantageous when the emission factor is <0.435 kg CO_2_e/(kW·h).^42^ Carbon emissions in silk production derive mainly from the use of electricity and heat in stages such as spinning, knitting, weaving, drying, boiling, dyeing, and finishing, often dependent on coal, gas, or wood, in addition to indirect emissions from auxiliary inputs. Shuyi et al.^43^ reported 18,563 kg CO_2_eq per 100 kg of silk products (cocoon to fabric); the UK organization, Waste & Resources Action Program (WRAP)^44^ estimated 21,820 kg CO_2_eq/tonne from fiber to garment; and Faragò et al.^45^ calculated 1820.97, 1,912.59 and 1,846.98 kg CO_2_eq per 100 kg of yarn-dyed, printed and open-width dyed silk fabrics, respectively. Considering the complete production inventories presented in Table 2, it can be observed that the climatic impact associated with silk, which includes stages such as cultivation, breeding and processing, among them degumming with sodium carbonate (Na_2_CO_3_) and boiling,^46,47^ typically ranges between 52.5 and 80.9 kg CO_2_e per kilogram of fiber, indicating that degumming contributes only a fraction of this total.^48^ Dissolution using lithium bromide (LiBr), due to the high energy cost of salt production and heating to around 60 °C, increases the footprint in kg CO_2_/kg fibroin.^49,50^ Purification processes, such as dialysis, filtration, and centrifugation, can further raise the impact of kg of CO_2_/kg of fibroin, depending on scale and efficiency. In contrast, water-based methods, which avoid the use of toxic solvents, tend to show lower emissions (kg CO_2_/kg fibroin), mainly due to reduced chemical use and more moderate energy consumption.^51^ Although these values provide a perspective, the literature often presents them in isolation, emphasizing the optimization of specific stages without adequately considering sustainability from a systemic standpoint. Thus, several studies focus on unit operations, neglecting the interactions among stages, such as degumming, dissolution, and purification. Furthermore, few analyses incorporate the social or economic dimensions of sustainability, such as occupational safety, solvent recovery costs, or industrial-scale feasibility.

**Table 2:** Processing of Silk Fibroin from the Cocoon for Manufacturing Biomaterials

parameters	degumming	dissolution	purification	water processing
Descriptions	Sericin removal	Fibroin solubilization	Impurity removal	Solvent-free fibroin recovery
Advantages	Simple, widely used	High purity	Improved quality	Eco-friendly
Challenges	May alter fibroin structure	Toxic solvents; high energy	Time-consuming; costly	Limited scalability
Applications	Raw fibroin extraction	Nanofibers, hydrogels	Biomedical devices	Biomanufacturing, biophotonics
CO_2_e Impact	Moderate	High	Moderate-high	Low
Key Impact	High energy demand (boiling); Na_2_CO_3_ use.	High energy demand; LiBr-based process	High water (dialysis); High energy (centrifugation)	Low chemical usage
refs	^52^,^53^	^54–56^	^57^,^58^	^59^,^60^

The widespread dependence on halide-based solvents such as LiBr illustrates this gap: despite well-known issues of toxicity and waste disposal, many studies continue to prioritize short-term efficiency over long-term environmental resilience.^61^ The literature also tends to treat “green” water-based processes as inherently sustainable, although these methods face significant challenges related to reproducibility, process control, and material quality at larger scales.^59^ Consequently, the notion of sustainability becomes relative, depending not only on CO_2_ metrics but also on broader environmental trade-offs associated with purity, durability, and biocompatibility. In the absence of standardized assessment frameworks, claims of “low-impact silk processing” risk being fragmented or inconsistent across studies. Future research should go beyond simple carbon accounting and aim for deeper integration among processing stages, redesigning degumming, dissolution, and purification as parts of a closed-loop, low-energy system rather than as isolated, energy-intensive steps. It is also necessary to invest in solvent innovation, exploring biobased or recyclable alternatives that can replace LiBr and other hazardous chemicals without compromising fibroin quality.^62^ Finally, the creation of standardized life-cycle assessment metrics and models is essential for consistently evaluating processing methods in both laboratory and industrial contexts, enabling fair comparisons and transparent sustainability measurements. Adopting this critical and holistic perspective brings silk biomaterial research closer to the United Nations Sustainable Development Goals (SDGs), particularly those related to responsible production (SDG 12), innovation (SDG 9), and health (SDG 3).^63–65^ Ultimately, achieving true sustainability in silk processing requires not only technical optimization but also a systemic re-evaluation of how materials, energy, and ethics intersect in biomaterial development.^66^ In this way, the methods of processing silk, described in Table 2, present different advantages and challenges with varying impacts on CO_2_. The widely used degumming process is effective in producing pure fibroin, but has a moderate impact due to energy use and chemical waste disposal.^67,68^ Dissolution, which offers high purity, uses toxic chemicals, which increases the carbon footprint. Purification improves material quality but is expensive and energy-intensive. Water processing, although more environmentally friendly, has scalability limitations and still requires energy. These processes highlight the need to seek more efficient and low-impact approaches. Adopting sustainable methods in the processing of silk biomaterials aligns with the SDGs, especially about health, innovation, and sustainability.^69^ Green processing techniques, which use renewable sources and low-energy technologies, help to create a greener industrial infrastructure and reduce carbon emissions. This not only contributes to human health by eliminating hazardous materials but also promotes more responsible production and consumption patterns, aligning with global efforts against climate change and favoring a more innovative and accountable industry.

## 6 Applications of SFb Nanomaterials

Derived from silkworm cocoons, the SFb is a nanomaterial with properties such as biocompatibility, biodegradability, and mechanical strength. SFbs can be selectively modified to achieve desired functional and structural properties.^70^ In biomedical applications, SFb can be used for drug delivery and tissue engineering.^71^ SFb nanoparticles carrying doxorubicin yield a targeted strategy for cancer therapy, and scaffolds inducing bone formation.^72–74^ In sustainable applications, silkworm domestication allows scaling-up of fibroin extraction. Recombinant DNA technology advances make fibroin production more environmentally friendly.^50^ Upcycling of biowaste from the silk industry, such as sericin, to produce hydrogels with antibacterial and antioxidative functionalities.^72^ SDG 3 (Good Health and Well-being): SFb’s applications include wound dressings, doxorubicin-loaded nanoparticles for cancer therapy, and bone repair scaffolds. SDG 9 (Industry, Innovation, and Infrastructure): SFb structural sensors, photonic devices, and flexible electronics replace synthetic plastics with biodegradable versions. It is a fibroin-polymer composite. SDG 12 (Responsible Consumption and Production): SFb reduces the environmental impact of manufacturing degradable fibers, films, aerogels, and hydrogels through water-based extraction.^75^ The bioproducts of the silk industry, sericin and cocoons, by converting them to nanoparticles, aerogels and hydrogels of high economic value, contributes to the circular economy.^76^ SDG 13 (Climate Action): The reduction of CO_2_ emissions and dependence on nonrenewable resources through the replacement of nonbiodegradable materials, such as in energy storage devices and textiles.^77–86^ SFb faces challenges in terms of scalability, cost efficiency, and quality consistency. Industrial extraction methods need optimization, and the protein’s intricate structure poses challenges for recombinant fibroin production. The SILK.IT project and life cycle assessments (LCA) as shown in Table 3. Recent studies have shown silk fibroin nanoparticles in drug delivery systems. For example, doxorubicin-loaded fibroin nanoparticles have shown an improved targeting efficiency and reduced side effects. Similarly, electrospun fibroin scaffolds have been successfully applied in bone tissue regeneration, demonstrating enhanced cell proliferation and mechanical stability.

**Table 3:**
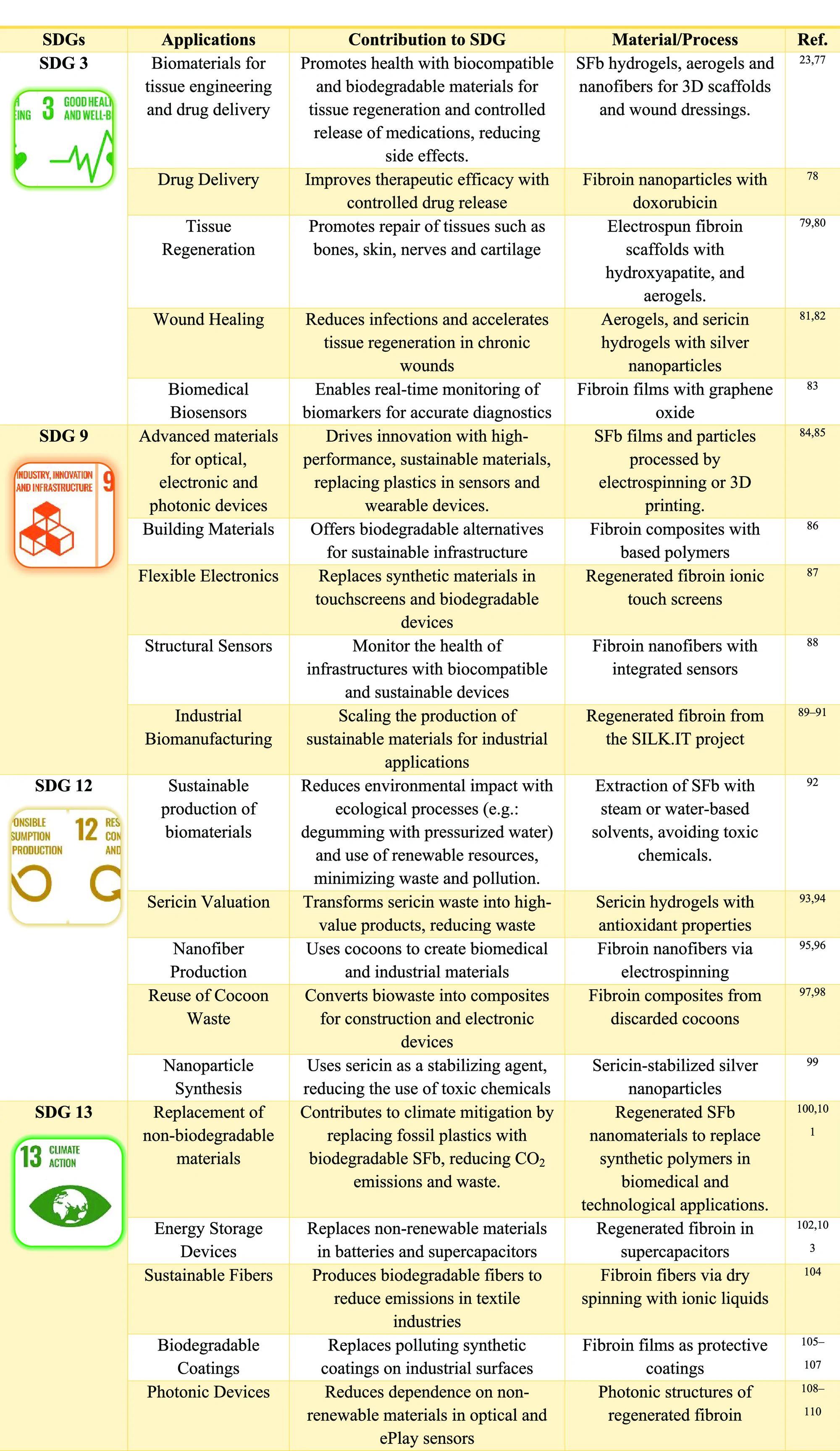
Applications of Silk-Fibroin Nanomaterials by SDGs

### 6.1 Sustainable Healthcare Solutions with SFb Nanomaterials

Silk-fibroin-based nanomaterials contribute to achieving SDG 3, which aims to ensure healthy lives and promote well-being for all ages. These materials are used for tissue regeneration, wound healing, and drug delivery. Using SFb’s biocompatibility, these developments in tissue engineering and drug delivery systems are shown in Figure 8a. These nanomaterials improve treatment-effect-controlled drug delivery systems, reducing side effects. SFb-based innovations improve our understanding of chronic diseases. Figure 8b shows SFb nanomaterials. Through microparticles, hydrogels, aerogels, nanoparticles, devices, and films, SFb drug delivery, wound healing, bone regeneration, and gene therapies. Bone regeneration and wound healing involve scaffolds (3D-supports), hydrogels, and films. The use of SFb nanomaterials for drug delivery involves controlled release systems as well as gene therapy. In tissue regeneration, SFb-derived materials are used in tissue engineering for regenerating cartilage, cornea, nerves, blood vessels, bones, and skin. These materials can be combined with other polymers to create scaffolds that support cell growth and tissue repair.^111^ SFb wound healing promotes tissue regeneration and reduces infection risks. It can be utilized in aerogels and nanoparticles.^112,113^ SFb’s capacity to encapsulate drugs and its drug-stabilizing properties prolonging shelf life and duration of action.^114^ The biodegradability of SFb enables therapeutic systems to be degraded within the body. The high compatibility of SFb and SFbs provides an environmentally friendly alternative to synthetic-based materials. It is well-suited for the development of eco-friendly biomedical devices.^115^ Scaling, cost, and regulatory considerations.

**Figure 8:**
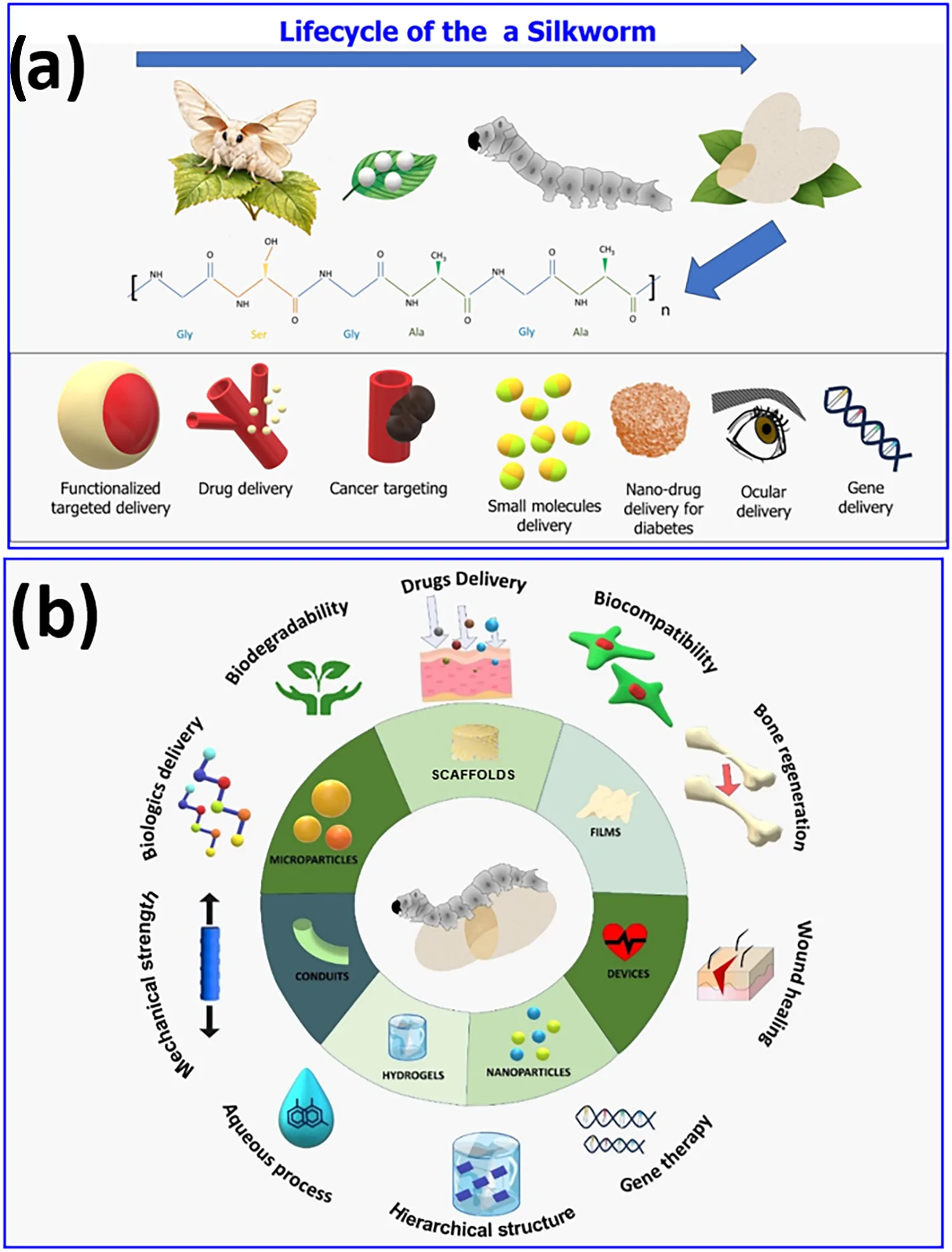
(a) Biomedical applications of silk fibroin-based nanomaterials. (b) Sustainable healthcare solutions with key silk-fibroin nanomaterials.

### 6.2 Silk Fibroin Nanomaterials for Industrial Applications

Silk fibroin is a sustainable nanomaterial. It is biocompatibility, biodegradability, and mechanical properties. SFb promotes the development of new materials.^116,117^ SFb can provide materials for durable infrastructure that reduces environmental degradation associated with construction.^117,118^ In the construction industry, SFb can be used to enhance mechanical properties and meet structural requirements, thereby improving the durability of infrastructure.^70,117^ In energy harvesting and electronics, SFb has properties, such as optical transparency and the ability to stabilize labile compounds, making it suitable for energy harvesting devices and electronic materials.^116,118^ In the fields of biodevices and sensors, the biocompatibility of materials and their capacity for controlled degradation are important for biosensors and biodevices.^118,119^ In flexible electronics, the creation of SFb ionic touch screens highlights their application in biodegradable devices.^87^ In the field of biodegradable coatings, SFb acts as a protective layer on surfaces.^118^ SFb nanomaterials face challenges such as production scaling and cost-effectiveness.^88,89^ SFb is employed in biomedical devices, such as electronic tattoos and wireless therapeutic systems, as well as sensors for real-time monitoring of pressure, temperature, and water, as shown in Figure 9a. It shows potential in energy technologies such as thermoelectric generators (TEG) and sustainable materials such as films and fibers for biomedical and industrial applications. SFb is a protein-based material with a molecular structure that allows for various modifications and processing techniques, enabling.^70^ Regenerated silk fibroin (RSF), produced through eco-friendly water-based processing, serves as a sustainable resource for applications in biomanufacturing and biophotonics.^90^ In industrial contexts, SFb is used in energy harvesting, electronics, and biomedicine.^91–101^ Its ability to be blended with other biobased materials is enhanced, making it suitable for multifunctional material design.^116^ The SILK.IT project scale-up SFb-based technologies from laboratory to industrial levels, focusing on sustainable manufacturing and advanced biotechnologies^90^ as shown in Figure 9b.

**Figure 9:**
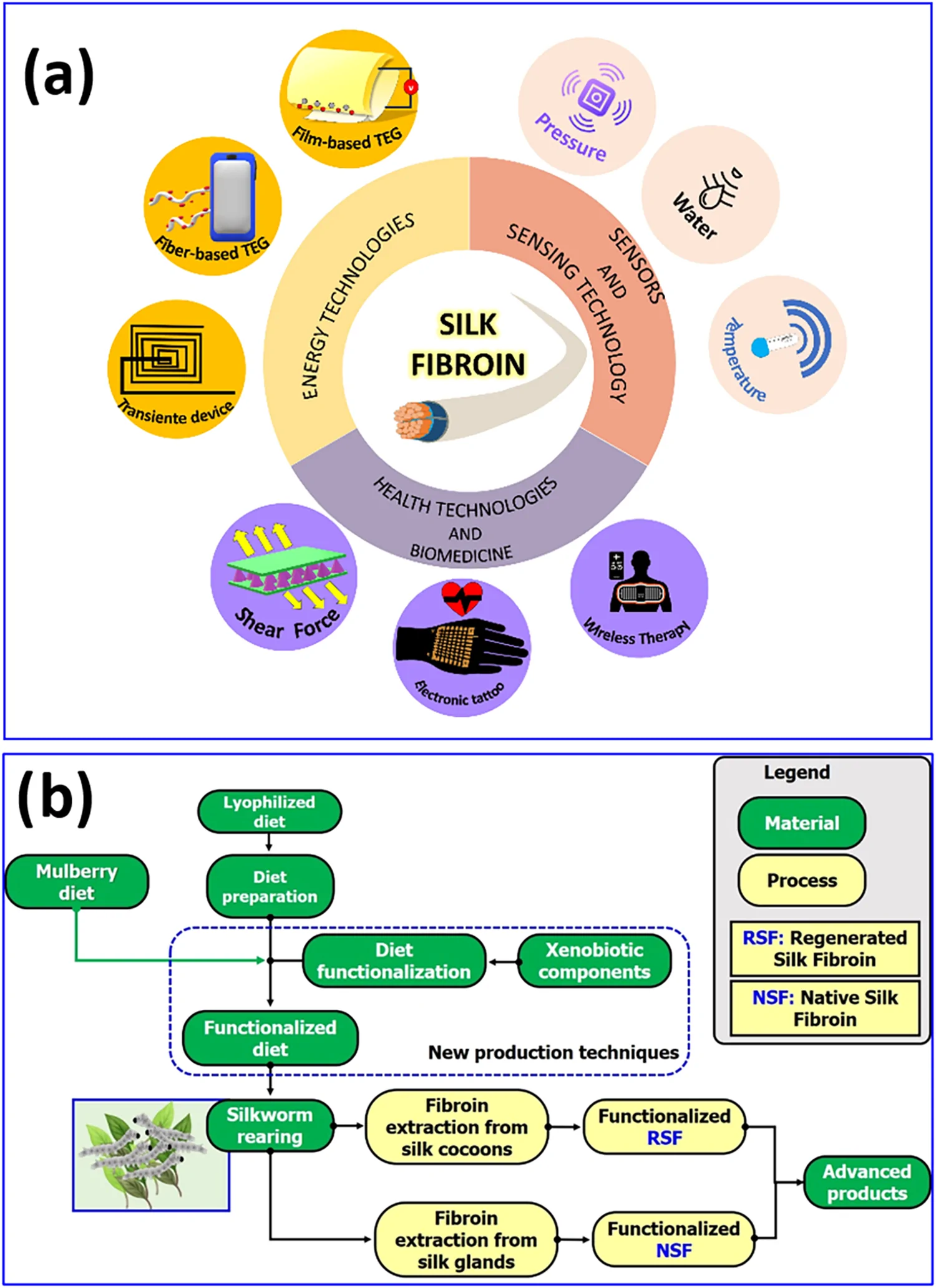
(a) Eco-friendly silk fibroin nanomaterials for industrial applications; (b) overview of the SILK.IT project concept and execution. Reproduced with permission from ref 90, Copyright (2015), Elsevier.

### 6.3 Valorization of Cocoons into High-Value Products

The development of SFb composites and blends has attracted attention.^116^ However, SFb presents challenges such as scalability and cost-effectiveness.^120^ The SFb and silk sericin in sustainable development^121^ mainly in intelligent wearables^122,123^ are shown in Figure 10a. Furthermore, the yarn produced from this waste can be used to create fabrics and other sustainable textile products.^124,125^ Silk fibroin and sericin, proteins from silkworm cocoons, are used in biomedicine, especially for wound healing and tissue regeneration.^126^ These cocoon-derived materials are also used in electronics, like electrodes.^127,128^ By applying functionalization techniques, their characteristics can be tailored to enhance durability and strength.^129,130^ These materials filtration systems, improving water purification and pollutant removal.^131–133^ Regenerated silk fibroin is a biodegradable material used in pharmaceuticals, cosmetics, and textile industries.^134,135^

**Figure 10:**
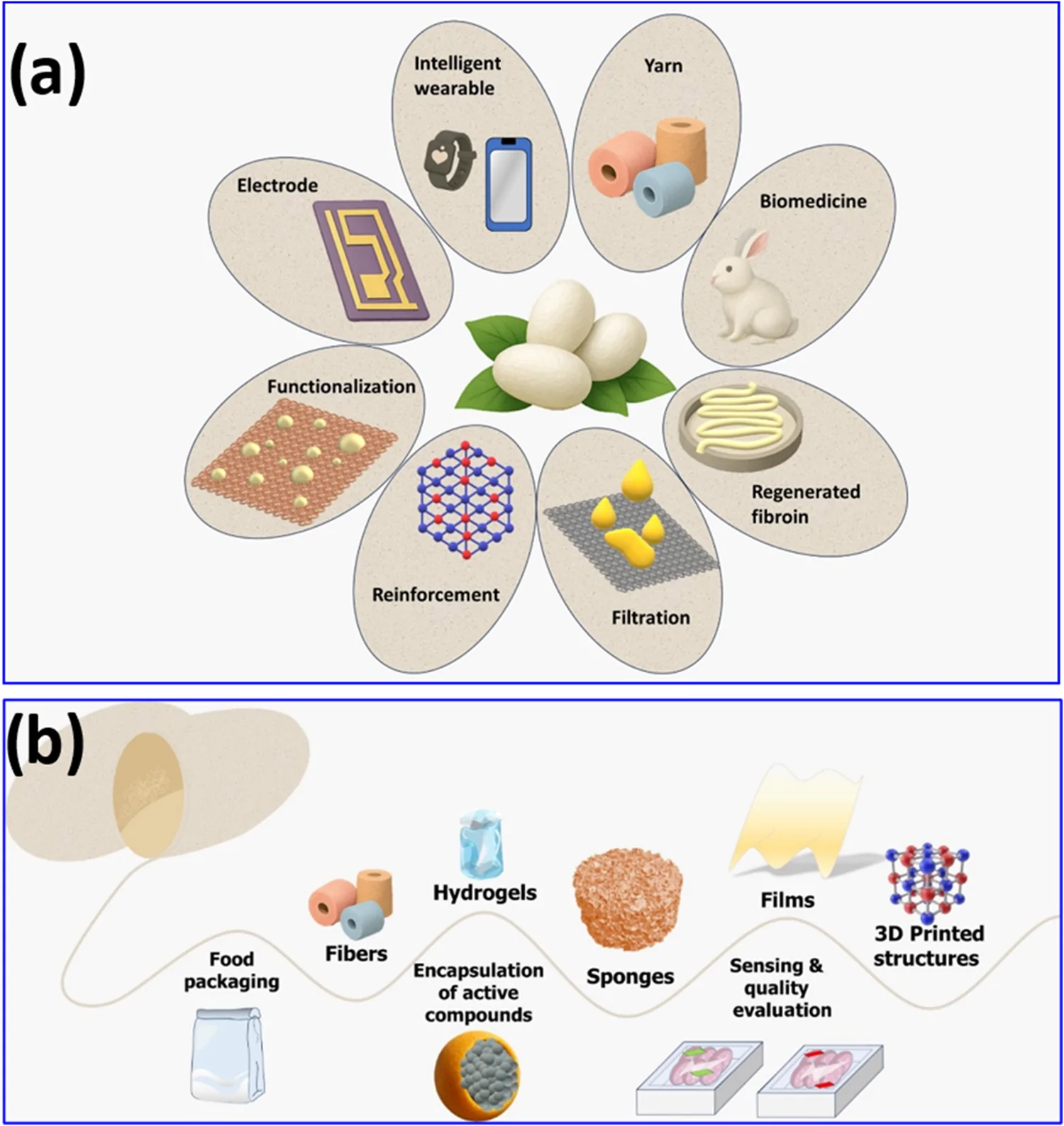
(a) Sustainable nanomaterials derived from silkworm cocoon for the development of valuable products. (b) Sustainable manufacturing initiatives in industrial-scale production of silk fibroin-based materials

Research has focused on sustainable nanomaterials from SFb and sericin in silkworm cocoons. These materials are studied for their properties and applications in biomedicine and nanotechnology. SFb is recognized for its biocompatibility, tunable degradability, and mechanical strength.^70,136^ Regenerated silk fibroin (RSF) can be produced through water-processing methods, reducing reliance on harmful chemicals.^90^ Approximately 50,000 tons of silk sericin are discarded annually, but it can be repurposed as a reducing and stabilizing agent in nanoparticle synthesis, such as silver nanoparticles.^137^ Sericin-based hydrogels have antibacterial and antioxidative properties.^72^ Projects like SILK.IT support industrial-scale production of SFb-based materials, promoting sustainable biomanufacturing practices.^90^ Challenges remain in scaling these technologies for widespread industrial application and ensuring economic viability. Silk fibroin, extracted from silkworm cocoons SFb, is utilized in various films, hydrogels, and nanoparticles. Its sensitivity and stability have also been investigated.^138^ Fibroin and sericin derived from cocoons create nanofibers and biomedical devices, as shown in Figure 10b. Sustainable SFb processing with water-based solvents minimizes environmental impact.^139^ The valorization of silkworm cocoon, sericin, regenerated fibroin, and/or cocoon residues is summarized in Table 4. Silver nanoparticles (AgNPs) and antioxidant hydrogels for wound dressing are synthesized with sericin. Similarly, water-based processed regenerated fibroin is transformed into nanofibers and films for biomedical and electronic applications. Furthermore, cocoon is recycled into a biocomposite for use in green building materials, resulting in a lower CO_2_ footprint.

**Table 4:** Valorization of Silkworm Cocoons

material	valuation method	high-value product	applications	sustainable impact	refs
Sericin 12.5% of the degumming wastewater with unused sericin was released	NPs synthesis	Silver NPs	Antibacterial dressings	Reduce the discard f coconuts&quot; appears as rom 50,000 tons/year to 400,000 tons/year in dried cocoons.	^140–142^
Sericin	Hydrogel formation	Hydrogels with antioxidant properties	Wound healing	Alternative to synthetic materials	^143^,^144^
Regenerated Fibroin	Water processing	Nanofibers, films	Biomedical devices, electronics	Eco-friendly process	^145–148^
Cocoon	Extraction and purification	Fibroin composites	Sustainable building materials	Reduces CO_2_ footprint	^149–152^

### 6.4 Challenges for Advancing S Fb Research

Nanomaterials were produced from SFb. Its applications include energy storage, electronics, and, in the field of energy storage and efficiency of SFb devices such as batteries and supercapacitors. In addition, the strong electrolyte interface and dendrite suppression by silk fibroin^153^ contribute to SFb-based supercapacitors, exhibiting energy-efficient wearable devices and smart sensors.^102^ SFb composites and blends of SFb with bio-based materials.^116,154^ Dry spinning of regenerated silk fibroin (RSF) using ionic liquids .^104^ SFb’s properties, such as biocompatibility and biodegradability, support its use in biomedical devices, optics, and electronics.^118^ Challenges persist in scaling their production and maintaining their cost-effectiveness. SFb enhances energy storage devices, such as batteries and supercapacitors. Its ability to form a solid electrolyte interface and prevent dendrite growth improves battery longevity and efficiency.^153^ SFb-based supercapacitors offer high performance and adaptability, ideal for wearable technology and energy-efficient smart sensors.^102^ SFb composites and blends with biobased materials improve processability and functionality, enabling the creation of sustainable alternatives to nonbiodegradable synthetics. This reduces carbon footprints across industries.^116,154^ Innovative techniques, such as dry spinning of regenerated silk fibroin (RSF) with ionic liquids, produce sustainable fibers for various applications while minimizing emissions.^104^ SFb’s biocompatibility and biodegradability make it suitable for biomedical devices, optics, and electronics, replacing environmentally harmful materials.^105–110^ These applications support sustainable development by reducing ecological impact.^118^ Despite these opportunities, challenges such as scalability and cost-effectiveness persist. Ongoing research and development are essential to optimize production processes and fully harness SFb’s potential for climate action and broader SDG alignment. A key challenge is scalability, as producing SFb on a large scale remains challenging, which limits its adoption in industry, as shown in Table 5. Process optimization occurs through initiatives like the SILK.IT project. Another issue is the high cost of extracting SFb, which restricts its economic viability. There has been variability in the quality of extracted fibroin. Standardizing purification protocols is recommended. Recombinant production is complicated due to the fibroin protein’s complex structure; however, advancements in recombinant DNA technology could facilitate its synthesis. SFb is biodegradable, but its long-term effects on the environment need to be studied. Life cycle analysis (LCA) is suggested.^155^

**Table 5:** Challenges and Future Perspectives in Silk Fibroin Research

challenges	descriptions	proposed solution	related research topic	refs
Scalability	Difficulty in producing fibroin on a large scale	Process optimization like SILK.IT	Sustainable biomanufacturing	^50^,^90^
Production Cost	Expensive processes limit economic viability	Use of eco-friendly methods (e.g., water)	Biowaste valorization	^156–158^
Consistent Quality	Variations in the quality of extracted fibroin	Standardization of purification protocols	Quality control in biomaterials	^23^,^61^,^159^
Recombinant Production	The complexity of protein structure hinders synthesis	Advances in recombinant DNA technology	Biotechnology for fibroin	^160–163^
Long-Term Environmental Impact	Limited data on large-scale degradation	Life Cycle Assessment (LCA)	Sustainability of nanomaterials	^48^,^164^,^165^

## 7 Challenges and Future Perspectives

This study explores nanobiomaterials and silk fibroin (SFb) nanoparticles for biomedical applications, as shown in Figure 11. **Theme 1** focuses on SFb and gold nanoparticles to improve the efficacy of chemotherapeutic agents.^166,167^ **Theme 2** explores nanostructured systems for drug delivery.^168^ **Theme 3** SFb drug delivery systems using microneedle technologies.^169,170^ **Theme 4** investigates SFb in tissue engineering and drug delivery,^171,172^ while **Theme 5** develops antibacterial nanomaterials, such as silver.^173,174^ Finally, **Theme 6** investigates multifunctional hydrogels for controlled drug release.^175^ **SDG 3:** SFb tissue engineering and drug delivery systems. **SDG 9:** SFb flexible electronics, sustainable construction materials, and advanced sensors. **SDG 12:** The use of SFb in biodegradable materials such as films, hydrogels, and aerogels (sericin and discarded cocoons). **SDG 13:** SFb helps reduce CO_2_ emissions and reliance on nonrenewable resources. Scalability and Cost: Scaling up production remains a challenge. Processing Complexity: The intricate molecular structure of SFb makes achieving a consistent quality difficult. Environmental Impact of Processing: Traditional silk fibroin production methods often involve toxic solvents and high energy consumption. Regulatory and Standardization Issues: The absence of standardized assessment frameworks complicates its commercialization. Life cycle assessments (LCA) are conducted according to regulatory standards.

**Figure 11:**
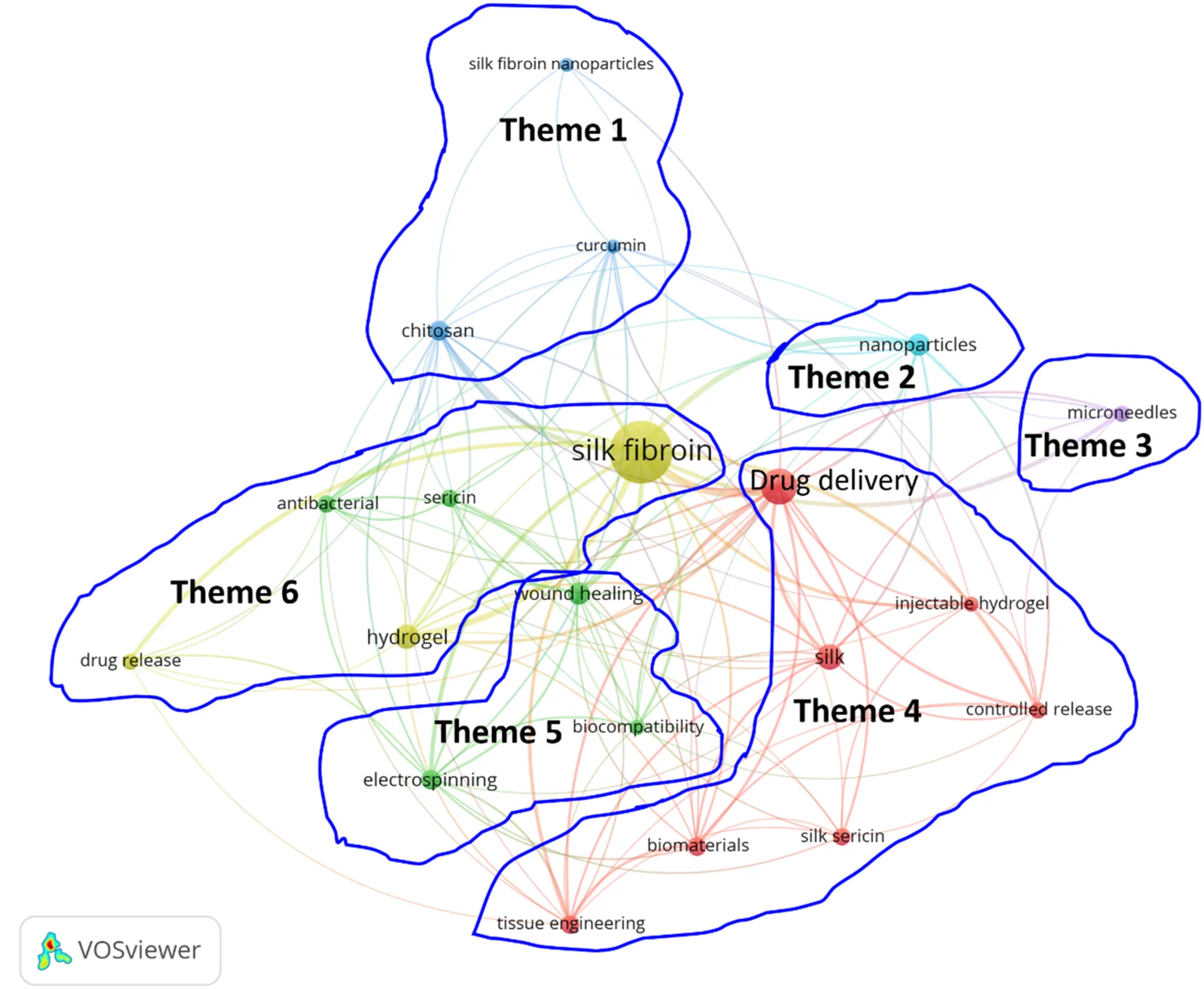
Analyzing keywords to create a thematic network map by VOSviewer (minimum 21 occurrences) in key research fields.

***Key findings***

- **Growth in Scientific Production:** Scientific publications on fibroin-based nanomaterials have experienced growth. China is the leading contributor, with significant contributions from Iran, the United States, and India. This research is concentrated in fields like Materials Science, Engineering, and Chemical Engineering.
- **Applications of SFb:** Silk fibroin-based materials have biomedical and industrial applications. In biomedicine, SFb is used for tissue engineering, drug delivery, wound healing, and bone regeneration. In industry, it is applied in sustainable construction materials, flexible electronics, and photonic devices.
- **Environmental Impact:** The production of SFb through traditional methods and water-based processing.
- **Valorization:** Silk fibroin and sericin are valuable byproducts of silkworm cocoon waste. These materials are aerogels, hydrogels, nanoparticles, and composites for medical and industrial use.
- **Alignment with SDGs:** SFb-based nanomaterials contribute to SDGs, SDG 3, SDG 9, SDG 12, and SDG 13.
- **Challenges and Future Directions:** While SFb, challenges persist in scaling production, optimizing processing, and ensuring cost-effectiveness. Advances in recombinant DNA technology, sustainable manufacturing, and LCA.

## 8 Conclusions

Research on nanomaterials based on SFb extracted from silkworm cocoons (*Bombyx mori*) has revealed a rapidly expanding field, with publications (66.44% between 2019 and 2025) and citations (74.84% in the same period), consolidating its scientific and technological relevance. Using a bibliometric-systematic approach with 1,791 articles analyzed in the Scopus database (2001–2025), highlights the versatility of fibroin in applications ranging from biomedicine, with advances in tissue engineering, controlled drug delivery, and wound healing, to industrial innovations, such as flexible electronics, sustainable building materials, sensors, and energy storage devices. These applications, driven by fibroin’s unique biocompatibility, biodegradability, and tunability properties, position it as a promising alternative to synthetic materials, directly aligning with SDG 3, SDG 9, SDG 12, and SDG 13. The valorization of silk industry waste, such as sericin and discarded cocoons, exemplifies a circular economy model, transforming approximately 50,000 tons of biowaste annually into high-value products, such as nanoparticles, aerogels, and hydrogels, with reduced CO_2_ impact in the SFb of the water-based processes. However, challenges such as scalability, production cost, and quality standardization persist, requiring innovations in sustainable biomanufacturing, such as the SILK.IT project, and advances in recombinant DNA technologies. Furthermore, assessing the long-term environmental impact through LCA is essential to maximizing the sustainability of these materials. This work highlights the transformative potential of SFb as a pillar for sustainable development, promoting innovative solutions that integrate health, technology, and environmental preservation. Continued research, focusing on ecological processes and global interdisciplinary collaboration, will be crucial to overcoming current barriers and consolidating fibroin as a key material in building a greener and more equitable future, in line with global sustainability goals.

## Supplementary Material



## Notes

Additional data for Methodological framework (CSV Scopus) for document retrieval, and overview of publications 2001–2025 (CSV)
